# Supplementary material for: Meisosomes, folded membrane microdomains between the apical extracellular matrix and epidermis
Source: eLife. 2023 Mar 13;12:e75906. doi: 10.7554/eLife.75906 (PMC10010689; doi:10.7554/eLife.75906)
Supplement: Supplementary file 1. [file elife-75906-supp1.docx]

| Strain | Genotype | Source |
| --- | --- | --- |
| IG274 | *frIs7[col-12p::DsRed, nlp-29p::GFP] IV* | (Pujol et al., 2008) |
| IG1697 | *dpy-2(e8) II; frIs7[nlp-29p::GFP, col-12p::DsRed] IV* |  |
| IG1685 | *dpy-3(e27) X; frIs7[nlp-29p::GFP, col-12p::DsRed] IV* | (Dodd et al., 2018) |
| IG1689 | *dpy-7(e88) X; frIs7[nlp-29p::GFP, col-12p::DsRed] IV* | (Dodd et al., 2018) |
| IG1699 | *dpy-8(e130) X; frIs7[nlp-29p::GFP, col-12p::DsRed] IV* | This study |
| IG344 | *dpy-13(e184) frIs7[nlp-29p::GFP, col-12p::DsRed] IV* | This study |
| IG1930 | *frEx624[pML670(VHA-5::GFP, unc-122p::GFP)]* | This study |
| IG1961 | *dpy-3(e27) X; frEx624[pML670(VHA-5::GFP, unc-122p::GFP)]* | This study |
| IG1990 | *pwSi46[hyp7p::mScarlet::HGRS-1,G418-R]; frEx624[pML670(VHA-5::GFP, unc-122p::GFP)]* | This study |
| IG2011 | *pwSi62[hyp7p::mScarlet::LGG-1,G418-R]; frEx624[pML670(VHA-5::GFP, unc-122p::GFP)]* | This study |
| IG2012 | *pwSi65[hyp7p::mScarlet::SNX-1,G481-R]; frEx624[pML670(VHA-5::GFP, unc-122p::GFP)]* | This study |
| IG2118 | *frSi26[pNP165(dpy-7p::VHA-5::GFP) ttTi5605] II* | This study |
| IG2129 | *frSi26[pNP165(dpy-7p::VHA-5::GFP) ttTi5605] II, ;pwSi46[hyp7p;;mScarlet::HGRS-1+G418-R]* | This study |
| IG2130 | *dpy-3(e27) X; frSi26[pNP165(dpy-7p::VHA-5::GFP) ttTi5605] II; pwSi46[hyp7p;;mScarlet::HGRS-1+G418-R]* | This study |
| PHX5715 | *vha-5(syb5715[VHA-5::sfGFP]) IV* | This study |
| IG2145 | *vha-5(syb5715[VHA-5::sfGFP]) IV x2* | This study |
| IG2144 | *dpy-7(e88) X; vha-5(syb5715[VHA-5::sfGFP]) IV x2* | This study |
| IG2128 | *mup-4(mc121[MUP-4::GFP]) III; mcIs52[VHA-5::mRFP1, unc-119(+)]* | This study |
| IG1809 | *dpy-2(e8) II; mup-4(mc121[MUP-4::GFP]) III* | This study |
| IG1813 | *frSi9[pNP151(col-62p::Lifeact::mKate_3'c-nmy), unc-119(+) ttTi5605] II; tbb-2(tj26[GFP::TBB-2]) III* | (Taffoni et al., 2020) |
| IG1814 | *dpy-3(e27) X; frSi9[pNP151(col-62p::Lifeact::mKate_3'c-nmy), unc-119(+) ttTi5605] II; tbb-2(tj26[GFP::TBB-2]) III* | (Taffoni et al., 2020) |
| IG1935 | *frSi9[pNP151(col-62p::Lifeact::mKate_3'c-nmy), unc-119(+) ttTi5605] II; Is[wrt-2p::GFP::PH-PLC1δ, wrt-2p::GFP::H2B, lin-48p::mCherry]* | (Taffoni et al., 2020) |
| IG2127 | *Is[wrt-2p::GFP::PH-PLC1δ, wrt-2p::GFP::H2B, lin-48p::mCherry],mcIs52[VHA-5::mRFP1, unc-119(+)]* | This study |
| PHX2235 | *rol-6(syb2235)[ROL-6::mScarlet]) II* | This study |
| IG2138 | *rol-6(syb2235[ROL-6::mScarlet]) II; Is[wrt-2p::GFP::PH-PLC1δ, wrt-2p::GFP::H2B, lin-48p::mCherry]* | This study |
| IG2137 | *dpy-3(e27) X; rol-6(syb2235[ROL-6::mScarlet]) II; Is[wrt-2p::GFP::PH-PLC1δ, wrt-2p::GFP::H2B, lin-48p::mCherry]* | This study |
| IG2136 | *rol-6(syb2235[ROL-6::mScarlet]) II; Ex[dpy-7p::GFP::CAAX_3'unc-54, myo-2p::GFP]* | This study |
| IG2135 | *dpy-3(e27) X; rol-6(syb2235[ROL-6::mScarlet]) II; Ex[dpy-7p::GFP::CAAX_3'unc-54, myo-2p::GFP]* | This study |
| IG2126 | *dpy-2(e8) II; Ex[dpy-7p::GFP::CAAX_3'unc-54, myo-2p::GFP]* | This study |
| MBA365 | *Ex[dpy-7p::GFP::CAAX, myo-2p::GFP]* | Michalis Barkoulas, UCL |
| ML2113 | *mcls67[dpy-7p::LifeAct::GFP; unc-119(+)] V; stls10088[hlh-1::his-24::mCherry, unc-119(+)]* | (Lardennois et al., 2019) |
| RT424 | *pwIs126[eea-1p::GFP::EEA-1]* | (Shi et al., 2009) |
| RT3657 | *pwSi46[hyp-7p::mScarlet::HGRS-1,G418-R]* | (Serrano-Saiz et al., 2020) |
| RT3640 | *pwSi65[hyp-7p::mScarlet::SNX-1,G481-R]* | (Serrano-Saiz et al., 2020) |
| RT3635 | *pwSi62[hyp-7p::mScarlet::LGG-1,G418-R]* | (Serrano-Saiz et al., 2020) |
| ML2771 | *mup-4(mc121[MUP-4::GFP]) III* | (Suman et al., 2019) |
| XW18042 | *qxSi722[dpy-7p::DPY-7::sfGFP; ttTi5605] II* | (Miao et al., 2020) |

**Supplementary Table S1**. Strains name & genotype with their associated references

Dodd, W., Tang, L., Lone, J. C., Wimberly, K., Wu, C. W., Consalvo, C., Wright, J. E., Pujol, N., & Choe, K. P. (2018). A Damage Sensor Associated with the Cuticle Coordinates Three Core Environmental Stress Responses in *C. elegans*. *Genetics*, *208*(4), 1467-1482. <https://doi.org/10.1534/genetics.118.300827>

Lardennois, A., Pasti, G., Ferraro, T., Llense, F., Mahou, P., Pontabry, J., Rodriguez, D., Kim, S., Ono, S., Beaurepaire, E., Gally, C., & Labouesse, M. (2019). An actin-based viscoplastic lock ensures progressive body-axis elongation. *Nature*, *573*(7773), 266-270. <https://doi.org/10.1038/s41586-019-1509-4>

Miao, R., Li, M., Zhang, Q., Yang, C., & Wang, X. (2020). An ECM-to-Nucleus Signaling Pathway Activates Lysosomes for *C. elegans* Larval Development. *Dev Cell*, *52*(1), 21-37 e25. <https://doi.org/10.1016/j.devcel.2019.10.020>

Pujol, N., Cypowyj, S., Ziegler, K., Millet, A., Astrain, A., Goncharov, A., Jin, Y., Chisholm, A. D., & Ewbank, J. J. (2008). Distinct innate immune responses to infection and wounding in the *C. elegans* epidermis. *Curr Biol*, *18*(7), 481-489. <https://doi.org/10.1016/j.cub.2008.02.079>.

Serrano-Saiz, E., Vogt, M. C., Levy, S., Wang, Y., Kaczmarczyk, K. K., Mei, X., Bai, G., Singson, A., Grant, B. D., & Hobert, O. (2020). SLC17A6/7/8 Vesicular Glutamate Transporter Homologs in Nematodes. *Genetics*, *214*(1), 163-178. <https://doi.org/10.1534/genetics.119.302855>

Shi, A., Sun, L., Banerjee, R., Tobin, M., Zhang, Y., & Grant, B. D. (2009). Regulation of endosomal clathrin and retromer-mediated endosome to Golgi retrograde transport by the J-domain protein RME-8. *The EMBO journal*, *28*(21), 3290-3302. <https://doi.org/10.1038/emboj.2009.272>

Suman, S. K., Daday, C., Ferraro, T., Vuong-Brender, T., Tak, S., Quintin, S., Robin, F., Grater, F., & Labouesse, M. (2019). The plakin domain of *C. elegans* VAB-10/plectin acts as a hub in a mechanotransduction pathway to promote morphogenesis. *Development*, *146*(24). <https://doi.org/10.1242/dev.183780>

Taffoni, C., Omi, S., Huber, C., Mailfert, S., Fallet, M., Rupprecht, J. F., Ewbank, J. J., & Pujol, N. (2020). Microtubule plus-end dynamics link wound repair to the innate immune response. *eLife*, *9*, e45047, Article e45047. <https://doi.org/10.7554/eLife.45047>
